# Clinical feasibility of MR-generated synthetic CT images of the cervical spine: Diagnostic performance for detection of OPLL and comparison of CT number

**DOI:** 10.1097/MD.0000000000025800

**Published:** 2021-05-07

**Authors:** Hee Seok Jeong, Chankue Park, Kang Soo Kim, Jin Hyeok Kim, Chang Ho Jeon

**Affiliations:** a Department of Radiology, Research Institute for Convergence of Biomedical Science and Technology, Pusan National University Yangsan Hospital, Yangsan; b Siemens Healthineers Ltd; c Department of Radiology, Pusan National University Hospital, Pusan, Korea.

**Keywords:** cervical spine, magnetic resonance imaging, ossification of posterior longitudinal ligament, sensitivity and specificity, synthetic computed tomography, ultra-short echo time

## Abstract

We aimed to determine the incremental value of magnetic resonance generated synthetic computed tomography (MRCT), evaluate cervical ossification of the posterior longitudinal ligament (OPLL), and compare the computed tomography (CT) numbers between MRCT and conventional CT.

Twenty-two patients who underwent magnetic resonance imaging (MRI) with MRCT protocols and CT were enrolled. MRCT images were generated from 3D-T2-weighted imaging, 3D-pointwise-encoding time reduction with radial acquisition, 3D-T1-Dixon, and 3D-time-of-flight sequences. Two radiologists independently evaluated the presence of OPLL at each cervical spine level during sessions 1 (MRI alone) and 2 (MRI + MRCT). CT was the reference standard for the presence of OPLL. One reader measured the mean CT number of the vertebral body and spinous process at each cervical spine level in the MRCT and CT images.

Sensitivity for the detection of OPLL was markedly higher in session 2 (MRI + MRCT) than in session 1 (MRI alone), as measured by both readers (47% vs. 90%, reader 1; 63% vs. 93%, reader 2). The mean CT number of MRCT and CT showed a moderate to strong positive correlation (ρ = .42–.72, *P* < .001).

The combined use of MRCT and MRI showed improved sensitivity for the evaluation of cervical OPLL. The mean CT number of MRCT and CT showed a positive correlation.

## 1 Introduction

Magnetic resonance-generated synthetic computed tomography (MRCT), also known as a pseudo- computed tomography (CT) image, involves segmentation of bone using voxel information from magnetic resonance imaging (MRI) and is represented as a CT-like image. Researchers have utilized the tissue characteristics that present in fat/water MR images to automatically segment various tissue types;^[1,2]^ however, separation of bone from air is challenging with conventional sequences owing to the short T2/T2∗ duration (0.4–0.5 ms). However, it has recently become possible to measure the CT number and visualize the bones using an ultra-short echo time (UTE) sequence with conventional sequences including the Dixon and time-of-flight (TOF) methods.

To date, the use of MRCT for radiation therapy in brain or head and neck oncology has been studied, with good inter-modality reliability for radiation therapy planning when compared to simulated CT.^[3–7]^ However, to our knowledge, no study has been conducted on the clinical application of MRCT imaging; therefore, we were interested in examining whether MRCT could be used to evaluate ossification, which is difficult to detect on MRI. Although studies have applied zero echo time (TE) images to osseous evaluation in the skull and temporomandibular joints,^[8,9]^ zero TE images are not real synthetic CT images. A zero TE image is just inverts the zero TE sequence image color, and the CT number cannot be measured.

Although MRIs are performed before cervical spine surgeries, a CT scan may also be performed to check for cervical ossification of the posterior longitudinal ligament (OPLL), osteophytes, or the bone margin. Of them, preoperative evaluation of OPLL is important because the approach method and postoperative prognosis vary depending on the presence and extent of the OPLL.^[10]^ We hypothesized that MRCT could reduce patients’ radiation exposure and additional examination time when it is used to aid OPLL evaluation. We aimed to determine the incremental value of MRCT for the evaluation of cervical OPLL and to compare the CT numbers of MRCT and conventional CT.

## 2 Methods

This retrospective study was approved by the institutional review board, and the requirement for informed consent was waived.

### 2.1 Study population

**Figure 1:**
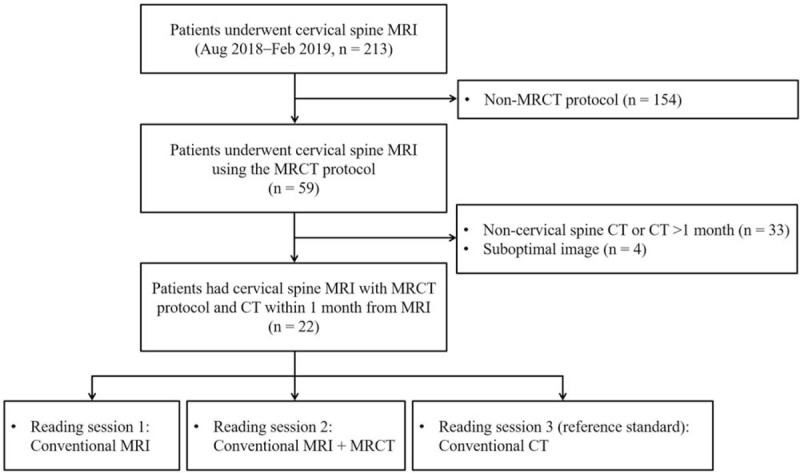
Flow diagram of the study patients. MRCT = magnetic resonance-generated synthetic computed tomography, MRI = magnetic resonance imaging.

Cervical spine MRI was performed for 213 adult patients between August 2018 and February 2019 at our institution. Of them, 59 patients underwent MRI using MRCT protocols; 33 of 59 patients did not undergo cervical CT or underwent CT more than 1 month after MR examination and were excluded. Four patients with suboptimal MR images were also excluded because of the presence of metal or motion artifacts. Thus, 22 patients were finally included in the study (Fig. 1).

Information regarding age, sex, and MRI and CT examination dates was obtained from the medical records.

### 2.2 Imaging technique

All patients received a 3.0-T unit MRI (Magnetom Skyra; Erlangen, Germany, Siemens Healthineers). All MRI examinations included conventional and MRCT protocols. Conventional protocols included a sagittal T1-weighted fast spin-echo (FSE) sequence (repetition time [ms]/echo time [ms]: 370–400/10–11) and a T2-weighted FSE sequence (3000–3290/85–109), with a 3-mm section thickness and a 250 × 250–260 × 260-mm field-of-view, and an axial T1-weighted FSE sequence (506–580/12–14) and a T2-weighted FSE sequence (7530–8710/90–106), with a 2-mm section thickness and a 140 × 140–150 × 150-mm field-of-view. MRCT protocols included three-dimensional (3D)-T2-weighted spin-echo-based sampling perfection with application-optimized contrasts using different flip angle evolutions (SPACE), 3D-pointwise encoding time reduction with radial acquisition (PETRA), 3D-T1-weighted volumetric interpolated breath-hold examination (VIBE) two-point Dixon, and 3D-TOF sequences. PETRA and TOF image volumes were acquired in the axial orientation, and T1-weighted VIBE Dixon and T2-weighted SPACE sequences were acquired in the sagittal orientation. The parameters for each MRCT sequence are presented in Table 1. Conventional CT examinations were performed with multiple products (Definition Flash and AS; Siemens Healthineers) using the following parameters: peak kilovoltage, 120 kV; tube current, 300 mA; gantry rotation time, 1.0 s; pitch, 0.8; and slice thickness, 2.0 mm. They were then reconstructed as axial and sagittal images.

**Table 1:** MR-generated synthetic CT imaging parameters.

	3D-T2WI	3D-PETRA	3D-T1 VIBE Dixon	3D-TOF
Repetition time (ms)	1200	3.3	5.7	19
Echo time (ms)	136	0.07	2.5	3.3
Flip angle (°)	140	6.0	10.5	15
Field-of-view (cm)	256 × 256	256 × 256	256 × 256	250 × 151
Matrix	320 × 320	320 × 320	320 × 320	320 × 116
Slice thickness/gap (mm)	1.0	0.8	1.0	1.0
Mean acquisition time	3 m 22 s	3 m 29 s	3 m 20 s	3 m 23 s

3D = three dimensional, CT = computed tomography, MR = magnetic resonance, PETRA = pointwise encoding time reduction with radial acquisition sequence, T2WI = T2-weighted imaging, TOF = time-of-flight, VIBE = volumetric interpolated breath-hold examination, WI = weighted imaging.

### 2.3 MRCT image reconstruction

**Figure 2:**
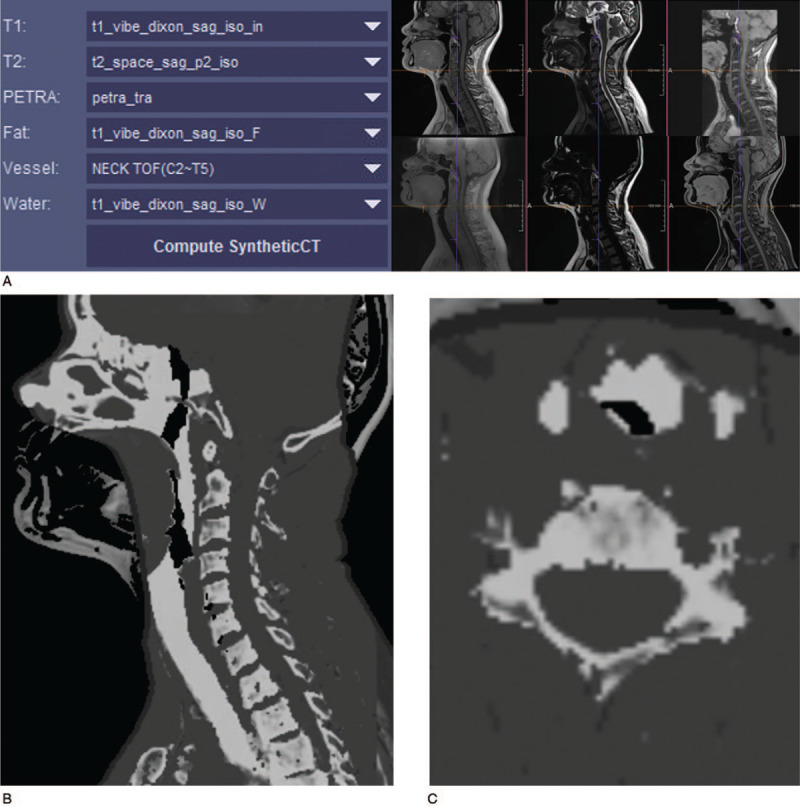
MRCT-image reconstruction process. A. For MRCT image reconstructions, we selected six sequences from four imaging parameters in the a syngo.via Frontier synthetic CT prototype: T1-weighted VIBE Dixon sequence was used for contouring of T1 contrast and for signal separation of fat and water. T2-weighted SPACE was used for contouring of T2 contrast and anatomical information. The role of PETRA was identification of air for defining an air mask to exclude such voxels from classification, and TOF was used to create a threshold mask to separate the blood flowing. B, C. Sagittal and axial MRCT images generated at approximately 5 min after pressing the run button. MRCT = magnetic resonance-generated synthetic computed tomography, PETRA = pointwise encoding time reduction with radial acquisition sequence, SPACE = sampling perfection with application-optimized contrasts using different flip angle evolution, TOF = time-of-flight, VIBE = volumetric interpolated breath-hold examination.

We reconstructed MRCT images using a syngo.via Frontier synthetic CT prototype (Siemens Healthineers). When we selected MRCT sequences in the program and pressed the run button, sagittal and axial MRCT images were generated (Fig. 2). The sequences for image reconstruction and their utilization were as follows: the T1-weighted VIBE Dixon sequence was used not only for contouring of T1 contrast but also for signal separation of fat and water. T2-weighted SPACE was used for contouring of T2 contrast and anatomical information (e.g., edema, nerve structure, and metastasis). PETRA was used to identify air for the purpose of defining an air mask to exclude such voxels from classification, and TOF was used to create a threshold mask to separate blood flowing. By expressing different MR contrasts in the above sequences, each voxel was classified by a fuzzy c-means clustering algorithm. A Hounsfield unit was assigned according to probability. The probabilities of voxels within each of the five classes (i.e., fat, fluid, gray matter, white matter, and bone) were determined using a fuzzy c-means clustering algorithm.^[11]^ Computation of the MRCT image took approximately 5 min.

### 2.4 Image analysis

#### 2.4.1 OPLL evaluation

Two radiologists (with 7 and 4 years of experience in musculoskeletal radiology, respectively) independently evaluated the presence of cervical OPLL at each cervical spine level (six segments: C1–2, C3, C4, C5, C6, and C7) in three separate reading sessions: 1) conventional MRI alone, 2) addition of MRCT to conventional MRI, and 3) conventional CT alone. To reduce recall bias, reading sessions for the same patient were conducted at least 1 month apart. We defined OPLL as a posterior ossification of the ligament behind vertebral bodies in sagittal images or characteristic upside-down T or bowtie configurations in axial images.^[12]^ In the third session, to obtain a reference standard value, the two readers independently analyzed the cervical OPLL on the conventional CT and, then, resolved the inconsistencies through a consensus review.

#### 2.4.2 Mean CT number measurement

**Figure 3:**
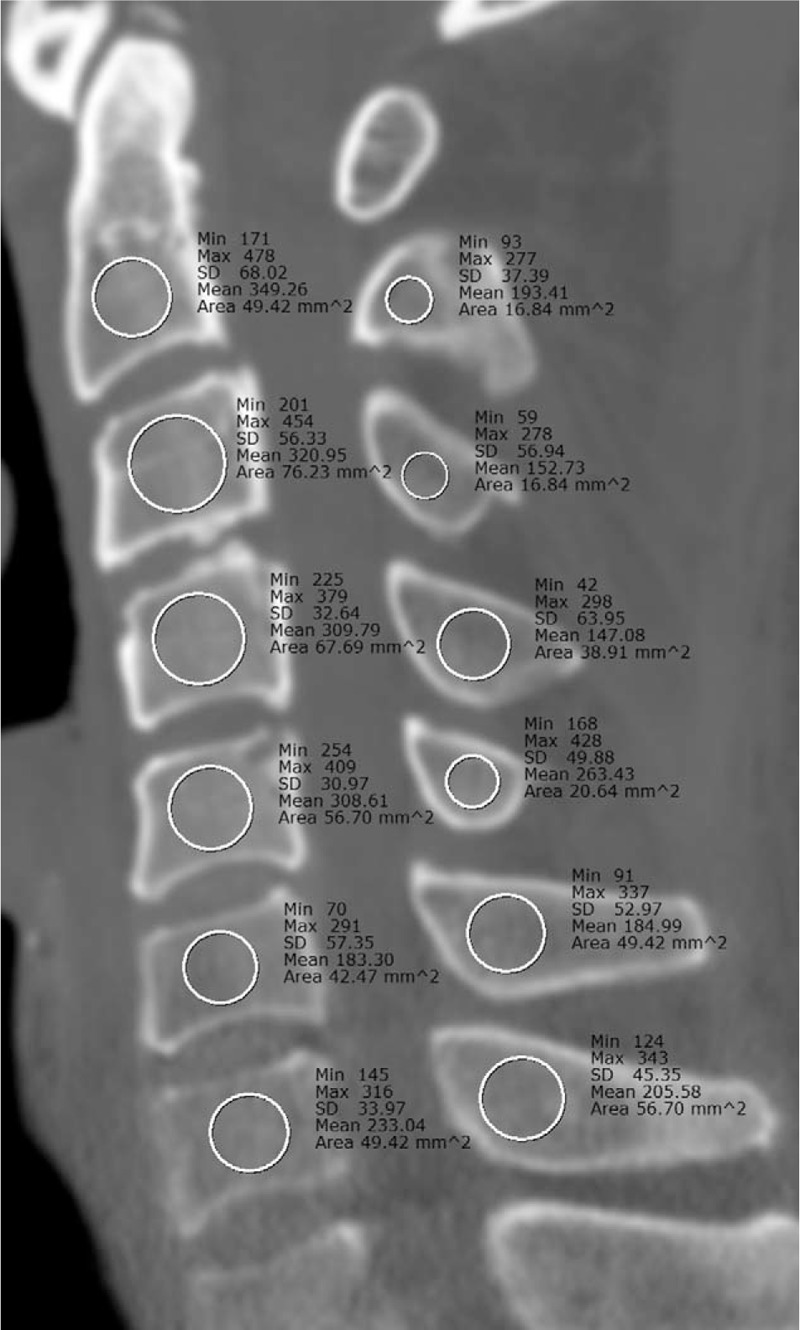
CT number measurement method. For comparison of the mean CT number between the MRCT and conventional CT images, the mean CT number (HU) at the midline of the vertebral body and spinous process at each cervical spine (six segments of C2–C7, a total of 12 measurements/pt) is measured using sagittal MRCT and conventional CT images. In the vertebral body, the circular ROI was manually positioned in the largest area of the bone not including the area of the cortical bone. HU = hounsfield unit, MRCT = magnetic resonance-generated synthetic computed tomography, ROI = region of interest.

One radiologist measured the mean CT number (hounsfield unit) at the midline of the vertebral body and the spinous process at each cervical spine level (six segments of C1–C7, for a total of 12 measurements/pt) in the sagittal MRCT and conventional CT images of all 22 patients (Fig. 3). In the vertebral body, the circular ROI was manually positioned in the largest area of the bone without including the area of the cortical bone. For the same measurement level, MRCT and conventional CT images were displayed side-by-side and then evaluated.

### 2.5 Statistical analysis

The sensitivity, specificity, positive predictive value, and negative predictive value were calculated based on a contingency table with data from conventional MRI in session 1 and data from conventional MRI with MRCT in session 2, with reference to data from conventional CT for the presence of cervical OPLL. Inter-reader reliability was assessed using a kappa (κ) statistic. The degrees of agreement based on κ values were interpreted using the following criteria: 0–.20, poor; .21–.40, fair; .41–.60, moderate; .61–.80, good; and .81–1.00, excellent. A simple correlation test was performed to assess the degree of correlation between the mean CT numbers of MRCT and conventional CT images. A p-value < .05 was considered to indicate statistical significance. Statistical analyses were performed using the statistical software SPSS version 26.0 (IBM, Armonk, NY, USA).

## 3 Results

We enrolled 22 patients in the study (mean age, 60.3 years; age range, 34–79 years). The sample comprised of 13 men (mean age, 61.3 years; age range, 34–79 years) and 9 women (mean age, 58.1 years; age range, 40–70 years). The mean time interval between MRI and CT examinations was 10.3 days (range, 0–29 days).

### 3.1 OPLL evaluation

**Figure 4:**
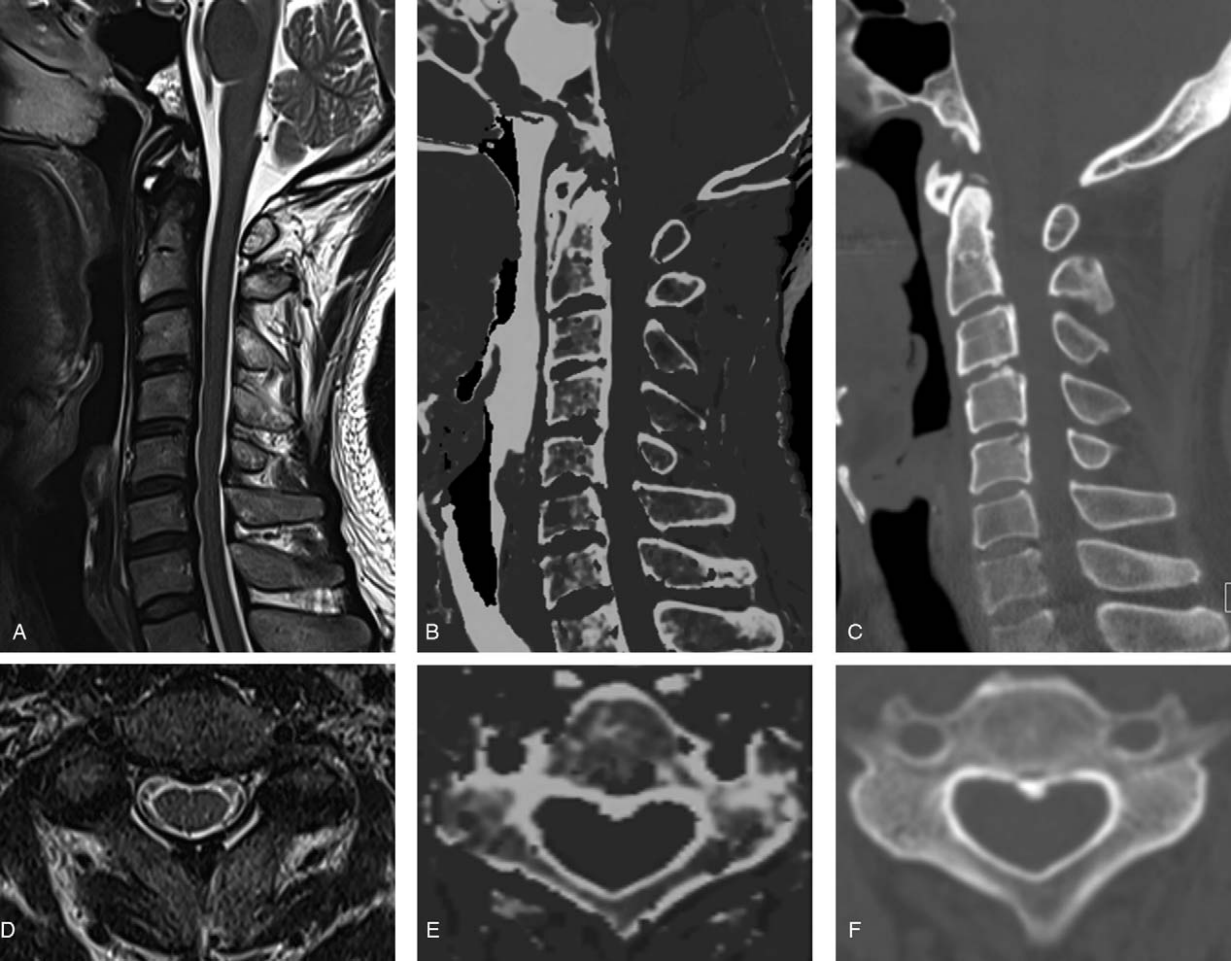
A 54-year-old-man with cervical OPLL.A, D. In session 1 (conventional MRI alone), two radiologists determined that there was thickening of cervical PLL but no OPLL at T2-weighted images. B, E. In session 2 (MRI + MRCT), two radiologists evaluated the presence of an OPLL at the C2–C5 level with the addition of MRCT to conventional MRI images. C, F. In session 3 (conventional CT), cervical OPLL was noted at the C2–C5 level, as the reference standard. MRCT = magnetic resonance-generated synthetic computed tomography, MRI = magnetic resonance imaging, OPLL = ossification of posterior longitudinal ligament, PLL = posterior longitudinal ligament.

The incidence of OPLL was 22.7% (30/132). Sensitivity for the detection of OPLL was markedly higher in session 2 (MRI + MRCT) than in session 1 (MRI alone) as measured by both readers (session 1 vs. 2: 47% vs. 90%, reader 1; 63% vs. 93%, reader 2). The specificity and positive predictive value were slightly lower and the negative predictive value was slightly higher in session 2 compared to that in session 1 (Table 2, Fig. 4). Inter-reader reliability was moderate (κ = .45) in session 1 and good (κ = .62) in session 2.

**Table 2:** Diagnostic performance of images for detection of the cervical OPLL.

Parameter	Sensitivity (%)	Specificity (%)	Positive predictive value (%)	Negative predictive value (%)
Reader 1				
Session 1	47 (29, 65) [14/30]	98 (92, 100) [100/102]	88 (60, 98) [14/16]	86 (78, 92) [100/116]
Session 2	90 (72, 97) [27/30]	89 (81, 94) [91/102]	71 (54, 84) [27/38]	97 (90, 99) [91/94]
Reader 2				
Session 1	63 (44, 80) [19/30]	94 (87, 98) [96/102]	76 (54, 90) [19/25]	90 (82, 95) [96/107]
Session 2	93 (76, 99) [28/30]	84 (75, 90) [86/102]	64 (48, 77) [28/44]	98 (91, 100) [86/88]

Data in parentheses present the 95% confidence intervals, and data in brackets present the numerator and denominator. Inter-reader reliability for the presence of OPLL was moderate (κ = .45) in session 1 (MRI alone) and good (κ = .62) in session 2 (MRI + MRCT). MRCT = magnetic resonance-generated synthetic computed tomography, MRI = magnetic resonance imaging, OPLL = ossification of the posterior longitudinal ligament.

### 3.2 Mean CT number measurement

The mean CT numbers of MRCT and conventional CT showed a moderate positive correlation for all segments (vertebral body + spinous process, ρ = .577, p < .001) and for the vertebral body (ρ = .420, p = .003), and a strong positive correlation for the spinous process (ρ = .716, p < .001).

## 4 Discussion

We investigated the diagnostic performance of cervical OPLL detection via MRCT images and found that this approach shows potential clinical applicability. When the MRCT images were added to the MRIs, the sensitivity of OPLL detection significantly increased, indicating that when cervical OPLL is actually present, there is an increased probability of detection by the examinations. This could reduce the incidence of cases in which OPLL is misdiagnosed as a thickening of the posterior longitudinal ligament in conventional MRIs. The mean CT number of MRCT and conventional CT showed a positive correlation, but there was a degree of difference by segment.

Many methods have been developed and investigated for generating MRCT images. Representative methods for MRCT imaging include bulk density override techniques, atlas-based techniques, and voxel-based techniques with UTE imaging.^[13]^ Voxel-based techniques combine information from multiple MRI contrasts, thus, resulting in a robust classification of tissue types.^[11]^ Voxel-based techniques with UTE have been shown to produce clinically acceptable geometric results. Therefore, we utilized a voxel-based technique with UTE and adapted it for applicability to the cervical spine. Additionally, the UTE-type protocol was necessary in order to distinguish bone and air. Among the UTE methods, we decided to use PETRA. Standard UTE is associated with streak artifacts that become more severe outside of the isocenter. In PETRA, data acquisition begins after a gradient ramp-up. To avoid a gap resulting in the center of the k-space, PETRA uses radial and cartesian sampling; the latter is used to fill the middle of the k-space. PETRA is a relatively reliable and clinically-released sequence, which also informed our selection of this sequence for UTE.^[13]^ Additionally, we used 3D distortion correction filters, as input images, to correct the field inhomogeneity of the PETRA images.

We conducted this study with the aim of reducing the need for additional CT examinations through evaluation using MRCT images before cervical operation. During this study, we experienced several issues that had to be overcome to achieve our goal. First, it took about 14 min to perform MRCT sequences; we found that applying a compressed sensing technique could reduce the examination time. Second, the main difference between MRCT and zero TE images was that MRCT could measure the CT number. In our study, the CT numbers of MRCT and conventional CT showed a strong positive correlation for the spinous process and a moderate positive correlation for the vertebral body. As the spinous process has a higher bone mineral density than that of the vertebral body,^[14]^ it is speculated that there may be a difference in the correlation of CT numbers due to the difference in bone density; further research is needed to prove this. Third, bone reconstruction was relatively successful using UTE, but a complete separation was not observed. As the program we used was an algorithm created by focusing on the head and the neck, masking may have been incomplete. If we create and use a classifying algorithm that applies only to the neck area in the future, masking may be improved. Although there were several limitations to the clinical application of MRCT, implementing CT images with MRI alone without radiation exposure is an attractive approach and better-quality imaging will be obtained as the physics of MRI continues to develop and more delicate classifying algorithms are applied in the future.

Our study had several limitations. First, we did not analyze the type of cervical OPLL. Second, as aforementioned, we reconstructed the MRCT image using an algorithm applied to the head and neck areas. If an algorithm focused on the neck classifier is developed and applied, it will produce more accurate masking and CT number correlation.

In conclusion, a combined analysis of MRCT and MRI was found to markedly improve sensitivity in the evaluation of cervical OPLL. The mean CT number of MRCT and CT showed a moderate to strong positive correlation.

## Author contributions

**Conceptualization:** Chankue Park, Kang Soo Kim.

**Data curation:** Chankue Park.

**Formal analysis:** Hee Seok Jeong, Chankue Park.

**Funding acquisition:** Chankue Park.

**Investigation:** Chankue Park, Jin Hyeok Kim, Chang Ho Jeon.

**Methodology:** Hee Seok Jeong, Chankue Park, Kang Soo Kim.

**Project administration:** Chankue Park.

**Supervision:** Chankue Park.

**Validation:** Hee Seok Jeong, Chankue Park, Kang Soo Kim, Chang Ho Jeon.

**Visualization:** Jin Hyeok Kim.

**Writing – original draft:** Hee Seok Jeong, Chankue Park, Kang Soo Kim.

**Writing – review & editing:** Hee Seok Jeong, Chankue Park, Jin Hyeok Kim, Chang Ho Jeon.
